# At-home sampling to meet geographical challenges for serological assessment of SARS-CoV-2 exposure in a rural region of northern Sweden, March to May 2021: a retrospective cohort study

**DOI:** 10.2807/1560-7917.ES.2023.28.13.2200432

**Published:** 2023-03-30

**Authors:** Julia Wigren Byström, Linnea Vikström, Ebba Rosendal, Remigius Gröning, Yong-Dae Gwon, Emma Nilsson, Atin Sharma, Akbar Espaillat, Leo Hanke, Gerald McInerney, Andrea Puhar, Felipe Cava, Gunilla B Karlsson Hedestam, Therese Thunberg, Tor Monsen, Fredrik Elgh, Magnus Evander, Anders F Johansson, Anna K Överby, Clas Ahlm, Johan Normark, Mattias NE Forsell

**Affiliations:** 1 Department of Clinical Microbiology, Umeå University, Umeå, Sweden; 2 Xerum AB, Umeå, Sweden; 3 Laboratory for Molecular Infection Medicine Sweden, Umeå University, Umeå, Sweden; 4 Department of Molecular Biology, Umeå University, Umeå, Sweden; 5 Department of Microbiology, Tumor and Cell Biology, Karolinska Institutet, Stockholm, Sweden

**Keywords:** Sweden, coronavirus disease (COVID-19), surveillance, laboratory

## Abstract

**Background:**

The current SARS-CoV-2 pandemic has highlighted a need for easy and safe blood sampling in combination with accurate serological methodology. Venipuncture for testing is usually performed by trained staff at healthcare centres. Long travel distances to healthcare centres in rural regions may introduce a bias of testing towards relatively large communities with closer access. Rural regions are therefore often not represented in population-based data.

**Aim:**

The aim of this retrospective cohort study was to develop and implement a strategy for at-home testing in a rural region of Sweden during spring 2021, and to evaluate its role to provide equal health care for its inhabitants.

**Methods:**

We developed a sensitive method to measure antibodies to the S-protein of SARS-CoV-2 and optimised this assay for clinical use together with a strategy of at-home capillary blood sampling.

**Results:**

We demonstrated that our ELISA gave comparable results after analysis of capillary blood or serum from SARS-CoV-2-experienced individuals. We demonstrated stability of the assay under conditions that reflected temperature and humidity during winter or summer. By assessment of capillary blood samples from 4,122 individuals, we could show both feasibility of the strategy and that implementation shifted the geographical spread of testing in favour of rural areas.

**Conclusion:**

Implementation of at-home sampling enabled citizens living in remote rural areas access to centralised and sensitive laboratory antibody tests. The strategy for testing used here could therefore enable disease control authorities to get rapid access to information concerning immunity to infectious diseases, even across vast geographical distance.

**Key public health message**

**What did you want to address in this study?**

Individuals with past SARS-CoV-2 infection have a higher degree of protection from severe COVID-19 after reinfection and better protection after vaccination. Although important, information was initially lacking on immunity in the population at large and in particular in remote areas. We wished to devise an easy strategy to accurately track which individuals produce antibodies against SARS-CoV-2 in a large human population.

**What have we learnt from this study?**

We find that the test results for seroconversion to SARS-CoV-2 following at-home sampling are comparable to the results obtained by regular sampling performed at a health clinic. We conclude that at-home sampling is convenient, easy to do by oneself and gives individuals living in rural areas the same access to the test as urban dwellers.

**What are the implications of your findings for public health?**

Our testing scheme enables public health authorities to get rapid information concerning immunity of the population to infectious diseases, even in remote areas. The methodology may be used not only for measuring the rate of infection in the community, but also if and how vaccines are effective in a given population.

## Introduction

The coronavirus disease (COVID-19) pandemic caused by the severe acute respiratory syndrome coronavirus 2 (SARS-CoV-2) has resulted in severe consequences for individuals and societies worldwide. Real-time monitoring of virus transmission has been achieved by extensive testing and analysis of virus RNA in respiratory samples. Additionally, as previous exposure reduces the risk of severe or fatal COVID-19 [1,2], serological studies designed to assess immune responses and durability of immunity in the population have also been of importance. Moreover, real-time epidemiological serosurveillance has been critical to support governmental decision-making during the pandemic [3]. 

Individuals who have been infected once with SARS-CoV-2 have a higher degree of protection from severe disease after reinfection than during primary infection [4]. Individuals who have had both a SARS-CoV-2 infection and have boosted immunity through vaccination manifested an even higher level of protection [5-7]. Accordingly, information of exposure to SARS-CoV-2 before vaccination can be used to estimate likelihood of severe disease in a population, and to devise and monitor strategies to reduce additional cases of severe COVID-19. This includes the allocation and prioritisation of healthcare resources and vaccine rollout [8-10]. Most patients with COVID-19 develop robust antibody responses to SARS-CoV-2 between 1 and 2 weeks following disease onset [11,12]. A large majority of all SARS-CoV-2 infections were asymptomatic or manifest with mild disease [13,14], and not all individuals developed strong and uniform antibody responses to all proteins of SARS-CoV-2. In addition, vaccine-induced immune responses to the spike (S)protein also forms the basis of currently used COVID-19 vaccines. As such, methodologies that measure antibodies towards the S-protein can be used both for serosurveillance of infections and for assessment of vaccine-induced responses.

Remote sampling has previously been used surprisingly rarely in the context of public health. The technology has been utilised to access and sample populations that are otherwise difficult to reach [15]. As such, remote sampling is thus very suited in geographical regions that are sparsely populated, with health care facilities located far from the inhabitants. The recent advent of quantitative dried blood spot (DBS) sampling kits makes large scale serological surveillance possible. The added advantages being that factors such as distances and climate may become irrelevant for sample acquisition. 

Here, we describe the development and evaluation of a sensitive assay that was optimised for detection of early or low-level anti-SARS-CoV-2 IgG responses using capillary dried blood spot (DBS) or venous blood samples. The assay was subsequently implemented in clinical practice at the University Hospital of Umeå, Sweden. Using a retrospective cohort study, we investigated the feasibility of remote sampling in combination with highly sensitive diagnostics, thereby providing equal care to all individuals of a sparsely populated geographic region of northern Sweden. Collectively, the findings reported here provide a blueprint for population-based immunosurveillance of SARS-CoV-2 exposure or vaccine coverage that is not limited by geographical constraints.

## Methods

### Study setting and population 

Region Västerbotten in northern Sweden comprises an area of 55,185 sq kilometers. This rural region is populated by 274,563 inhabitants (source: www.regionfakta.com on December 2021), of whom more than 200,000 live in one of two urban areas, Umeå or Skellefteå. Three hospitals (University Hospital of Umeå, Skellefteå Hospital, Lycksele Hospital), 33 regional primary healthcare centers and four privately run primary healthcare centers serve the population. Health services are tax-funded in Västerbotten, as throughout Sweden. 

This study takes advantage of a Swedish government mandate [16] that allowed voluntary antibody testing for COVID-19 of the public at a subsidised price, organised by the regions in Sweden. In Region Västerbotten, all residents aged >16 (ca 220,000 individuals) were eligible to do an antibody test, using two different sampling methods at two separate time intervals. Between November 2020 and January 2021, venous blood samples were taken by phlebotomists at select sites in Umeå and Skellefteå, and by transient ambulatory teams outside of these areas. Between March and May 2021, residents could order an antibody test online, and a capillary blood sampling kit would be sent to their home for self-sampling. allowing increased access to rural regions. The accessibility of testing between the two periods was compared to examine the propensity of at-home sampling after the shift of COVID-19 testing to rural regions.

The COVID-19 vaccine roll-out was ongoing in Sweden during the study period, starting in December 2020 with the oldest individuals living at nursing homes, i.e. people aged 80 years and above, and healthcare staff, continuing with individuals aged >70 and risk groups and subsequently individuals aged >18 [17]. At the end of the study period (May 2021), about 20% of the residents in Region Västerbotten aged 18–59 years were vaccinated with the first dose (personal communication Therese Thunberg, Västerbotten, Centre of Disease Control). 

### Venous and capillary blood sampling for public antibody testing

The majority of serum or capillary samples used for this study were part of the voluntary public antibody testing for COVID-19 collected by the University Hospital of Umeå for clinical purposes within the realm of providing healthcare in Region Västerbotten, Sweden. 

Venous blood samples were collected by phlebotomy between 16 November 2020 and 21 January 2021 in accordance with clinical practice. The residents could book an appointment for sampling at select sites in Umeå and Skellefteå. Transient ambulatory teams performed sampling outside of these areas. 

Capillary blood samples were collected by self-sampling between 1 March and 18 May 2021. An antibody test could be ordered on-line by the residents and capillary blood sampling kits were sent by mail to their home. A sampling kit comprised a quantitative DBS sampling card (qDBS, Capitainer AB, Stockholm, Sweden), a contact-activated lancet (BD Microtainer #366594), a return pouch, an alcohol swab, gauze, self-sampling instructions, a sample bar code and a pre-paid return envelope. The qDBS sampling card comprised two filter discs for collection of 10 µl capillary blood each, according to the manufacturer’s instructions (Capitainer AB). At least one of two filter paper discs filled with 10 µl blood was needed to conduct the following antibody analysis. Venous blood samples and qDBS samples were returned to the Clinical Microbiology Laboratory at the University Hospital of Umeå, and samples were analysed for SARS-CoV-2 N- and S-specific IgG. 

Test results were returned to the individual under confidentiality in accordance with clinical practice. Anonymised demographic data for evaluation of the at-home sampling or venous blood sampling strategies were provided by Region Västerbotten.

### Assay development

#### Venous and capillary blood samples for assay development

For SARS-COV-2 S ELISA assay development, we used blood samples that were collected for clinical purposes at the University Hospital of Umeå. We used 30 serum samples from 25 symptomatic hospitalised COVID-19 patients with qPCR-confirmed SARS-CoV-2 infection sampled 2–30 days post-symptom onset. For comparison, we also used 144 serum samples from anonymised individuals who had been diagnosed with other coronavirus infections, other virus infections or rheumatoid arthritis before the emergence of SARS-CoV-2. For comparison between the S ELISA and N-directed Abbot Architect SARS-CoV-2 assays and the LIASON SARS-CoV-2 S1/S2 assay (DiaSorin, Inc.), we used a subset of the previously mentioned pre-pandemic (n = 30) and the qPCR-confirmed SARS-CoV-2 (n = 23) serum samples used in the S ELISA validation. For further comparison between S ELISA and Abbot Architect SARS-CoV-2 assay, we used 145 anonymised samples that had been collected by the University Hospital of Umeå for the purpose of infection prevention.

#### Protein production

A plasmid encoding the 2019-nCoV S protein was kindly provided by Jason McLellan and has been previously described [18]. Protein was produced using the Freestyle MAX 293 Expression System (ThermoFisher Scientific). Briefly, plasmids encoding the SARS-CoV-2 S protein (Wuhan strain) was transfected into the cells using Freestyle MAX reagent in a 1x10^6^ cells/ml culture and grown for 4–5 days in 8% CO_2_ and 120 rpm. The supernatant was then cleared of cells and debris by centrifugation and by passage through a 0.2 µM filter. The supernatant was then flowed over column packed with His-pure Ni-NTA resin (ThermoFisher Scientific) at a rate of ca 0.5ml/minute. Subsequently the column was washed with 10 column volumes of 20mM imidazole/phosphate-buffered saline (PBS) pH 7.4 and then eluted with 250 mM imidazole/PBS pH 7.4. The resulting eluate was concentrated, and buffer exchanged to PBS pH 7.4 by Amicon spin columns with a cut off < 100 kDa (Sigma Aldrich). In an alternative set-up for purification of S protein for competition assays, the S protein was first captured via glycans in lentil-lectin chromatography (GE Healthcare), washed with PBS and then eluted with 0.5 M methyl-α-D-mannopyranoside before loading of the elute on a nickel-nitrilotriacetic acid (Ni-NTA) packed column. Protein purity was determined by SDS-page, native gel electrophoresis and concentration was determined by a BCA Protein Assay kit (ThermoFisher Scientific).

#### SARS-CoV-2 spike ELISA assay

In this study, we used two SARS-CoV-2 spike ELISA protocols. For both assays, clear flat-bottom Immuno MaxiSorp 96-well plates (Thermo Scientific) were coated with 200 ng/well of purified SARS-CoV-2 S protein and incubated at + 4°C overnight. The following day, wells were washed once with PBS-0.05% Tween (PBS-T) and incubated for 1 h at room temperature (RT) with blocking buffer (1% non-fat dry milk in PBS-T). In the alkaline phospahate (AP) S ELISA protocol, duplicates of human serum samples were diluted 1/100 in blocking buffer and added to the ELISA plate. For each plate, control serum samples from a highly anti-S IgG-positive individual, and blank wells with blocking buffer alone was also included. Subsequently, the plate was incubated for 1 h at RT and then washed four times with PBS-T. Goat-anti human IgG AP-conjugated antibody (Thermo Scientific) was diluted 1/6,000 in blocking buffer and 100 µl was added to each well and incubated for 1 hour at + 37 °C. The wells were then washed four times with PBS-T, followed by addition of 100µl/well of AP colourimetric substrate containing 1 mg/ml phosphatase substrate (Sigma-Aldrich) dissolved in diethanolamine buffer. The plate was incubated at + 37 °C for 30 min, and the reaction was stopped with 50 µl 3 M NaOH. Detection of anti-S IgG was then analysed at 405nm with the Tecan Sunrise reader. The final optical density (OD) was calculated as OD_405nm_(sample) − OD_405nm_ (blank well). In some instances, we used a horseradish peroxidase (HRP)-conjugated anti-human IgG as secondary antibody (Thermo Scientific) at a dilution of 1/5,000 dilution in blocking buffer. Colourimetric change using the 1-Step Ultra TMB-ELISA (Thermo Scientific) was measured at 450nm after 2M H2SO4 had been added to stop the reaction. Because of the relatively rapid colourimetric change and increased background by the HRP-conjugate in comparison to the AP-conjugate, the latter was deemed more suitable for the large-scale sampling strategies described herein.

#### Spike competition assay

A spike protein competition assay was performed to verify inconsistent positive SARS-CoV-2 antibody results that differed between the S ELISA and the Architect or Liaison instruments. Serum was diluted 1/10 in PBS and purified S protein (100 µg/ml) or mock (PBS) was added and incubated for 1 h at RT. The serum/S protein mixture was diluted 10-fold with blocking buffer to obtain a final serum dilution of 1/100 and 100 µl per well was added in duplicates to an S ELISA plate.

#### Virus and plaque neutralisation assay

Vero E6 cells were cultured in Dulbecco’s modified Eagle’s medium (DMEM; D5648 Sigma-Aldrich) supplemented with 5% fetal bovine serum (FBS; HyClone), 10 units/ml penicillin and 10 µg/ml streptomycin (PeSt, HyClone). The patient isolate SARS-CoV-2/01/human/2020/SWE (GenBank accession: MT093571.1) was provided by the Public Health Agency of Sweden, Stockholm. The viral stock was grown in VeroE6 cells for 48 h and titrated by a plaque assay. Briefly, VeroE6 cells (4x10^5^/well) were seeded in 12-well plates 12–24 h before infection, and a 10-fold serial dilution of virus was added. After 1 h, 2 ml semisolid overlay containing DMEM + 2% FBS + PeSt + 1.2% Avicel RC/CL was added and cells were incubated at 37 °C in 5% CO_2_. After 65 h, the overlay was removed and cells were fixed with 4% formaldehyde for 30 min, washed with PBS and stained with 0.5% crystal violet in 20% MeOH for 5 min. Plates were washed with water and the plaques were counted. To perform a neutralisation assay, serum samples were heat-inactivated at 56 °C for 30 min and 10-fold dilutions of sera were prepared in virus stock (250 PFU/ml in DMEM + PeSt). The virus/sera mix was incubated at 37 °C in 5% CO_2_ for 1 h, then 400 µl virus/sera inoculum were added to VeroE6 cells and the plaque assay performed as described above.

#### Fluorescent inhibition assay

Vero E6 cells (10^4^/well) were seeded for 12–24 h in 96-well plates (Greiner CELLSTAR) before infection. Heat inactivated serum samples were diluted 1:10 in virus solution (10^4^ PFU/ml in DMEM + PeSt) and then serially diluted fivefold in the same virus preparation. The virus/sera mix was incubated at 37 °C in 5% CO_2_ for 30 min, then 50 µl virus/sera inoculum was added to the cells and incubated for another 2 h at 37 °C in 5% CO_2_. The inoculum was removed and 100 µl media containing DMEM + 2% FBS + PeSt were added and the cells were incubated at 37 °C in 5% CO_2_. Eight hours post-infection, cells were prefixed by replacing 50 µl of media with 4% formaldehyde for 10 min at RT and fixed for 30 min in 4% formaldehyde. Plates were washed with PBS, permeabilised with 0.5% Triton X-100 in PBS and 20 mM glycine for 10 min at RT, followed by blocking with PBS containing 2% BSA for 30 min at RT. Virus-infected cells were stained for 1 h with anti-SARS-CoV-2 N protein rabbit monoclonal antibody (40143-R001, Sino Biological) diluted 1:1,000 in blocking buffer, followed by secondary donkey anti-rabbit IgG (H + L) Alexa Fluor 488 antibody (Invitrogen) 1:1,000 in blocking buffer for 30 min and DAPI staining (0.1 ug/mL in PBS) for 5 min. The number of infected cells was quantified using a TROPHOS Plate RUNNER HD (TROPHOS SA, Marseille, France).

### Clinical assay validation of capillary blood testing

#### Venous and capillary blood sampling for capillary blood test validation

We used paired serum and qDBS samples from 149 individuals collected by the University Hospital of Umeå, and the Department of Clinical Microbiology, Umeå University, Umeå, Sweden for capillary blood test validation. Sixty-three of these individuals were asked to answer a questionnaire about how they experienced the capillary blood self-sampling procedure.

#### Dried blood spot sample preparation

After sampling, 10µl of whole blood was added to the two paper discs on the qDBS, sample card (Capitainer AB). The discs were extracted with a semi-automated puncher (Capitainer AB) and placed in a single well of a 96-well plate. Blood components were eluted by adding 100 µl PBS-T containing protease inhibitor cocktail (#4693116001, Roche) and subsequent incubation in a shaker (170 rpm) for 1 h in RT. Blood eluate (20 µl) was mixed with 80 µl blocking buffer and analysed in duplicate for SARS-CoV-2 S-directed IgG at a final dilution of 1/50.

#### Stability test for seasonal changes

Anonymised positive and negative control human EDTA plasma samples with known S-directed IgG levels [19] were received from Karolinska University Hospital, Stockholm, Sweden. In total, two negative samples, four low positive, two moderate positive and two high positive samples were obtained. Whole blood from a healthy donor negative for S-directed IgG was mixed with the aforementioned plasma samples to a haematocrit of 43%, producing whole blood with different levels of anti-S-IgG reactivity. Spiked blood with negative (n = 30), low (n = 60), moderate (n = 30) or high (n = 30) S-directed IgG was then applied to qDBS sampling cards, which were divided into three identical groups with 50 sampling cards each. The qDBS sampling cards were placed in paper envelopes and incubated at RT or in a climatic chamber (MHK-800 YK, Terchy Environmental Technology Ltd.) with two different temperature and humidity scenarios to reflect shipping of sample cards under winter or summer conditions, as specified by United States Food and Drug Administration (FDAs) guidance document on home specimen collection serology template for fingerstick DBS samples [20]. The 56-h summer profile consisted of the following temperature steps: 8 h at + 40 °C, 4 h at + 22 °C, 2 h at + 40 °C, 36 h at + 30 °C, 6 h at + 40 °C and the humidity was set to a constant 75% relative humidity (RH). The 56-hour winter profile consisted of the following temperature steps: 8 h at −10 °C, 4 h at + 18 °C, 2 h at −10 °C, 36 h at + 10 °C, 6 h at −10 °C and the humidity was set between 10 and 40% RH. After the qDBS sampling cards been exposed to RT or the two different temperature and humidity cycling programs, the samples were shipped in boxes by regular courier transportation to the laboratory of the Department of Clinical Microbiology at Umeå University, Umeå, Sweden. The shipment took 4 days. During this time, the boxes were handled as a standard package by the courier company at ambient temperature (November 2020). Upon arrival at the laboratory, the samples were analysed for SARS-CoV-2 IgG using the AP S-ELISA.

### Statistical analysis

Statistical analysis was performed by using Prism 8 (GraphPad Software). We used non-parametric Kruskal-Wallis test for comparisons and Dunn’s multiple comparison test for post-hoc analyses. Correlative analysis was done by calculation of the Spearman r correlation.

## Results

### Validation of a SARS-CoV-2 spike protein-based assay for detection of early seroconversion 

**Figure 1:**
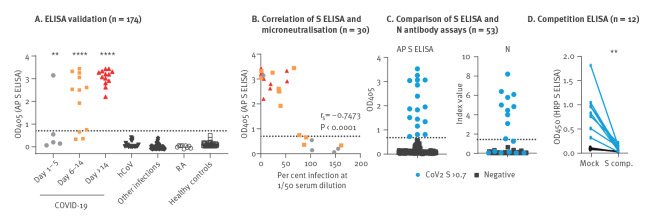
Validation of ELISA methodology to detect binding and neutralising anti-SARS-CoV-2 S-specific IgG, Region Västerbotten, Sweden, March–May 2020

COVID-19: coronavirus disease; CoV-2: coronavirus 2; hCoV: human coronavirus; OD: optical density; RA: rheumatoid arthritis; S: spike protein; N: nucleocapsid protein; SARS-CoV-2: severe acute respiratory syndrome coronavirus 2.

To develop a sensitive assay to detect anti-SARS-CoV-2 S IgG from venous blood, we produced recombinant trimeric S proteins (S-2P) from the SARS-CoV-2 isolate Wuhan Wu-1 [21] and used these as antigens to develop an ELISA-based set-up for detection of anti-S-directed IgG in serum or plasma of SARS-CoV-2 infected individuals (S-ELISA). After confirmation that we could detect anti-S IgG in a concentration-dependent manner (serum titration shown in Supplementary Figure S1A shows IgG levels in serum from COVID-19 patients or none-exposed individuals at different dilutions), we proceeded to define a cut-off for positive signal when used in single dilution-point format against 144 pre-pandemic samples and 30 samples from hospitalised patients with qPCR-confirmed SARS-CoV-2 infection. The mean OD value of pre-pandemic samples was 0.06 +/− 0.12 and we set a stringent cut-off for positive detection of S-specific IgG to OD 0.7 or higher (OD: 0.06; SD: 5.8). By applying this cut-off, we detected seroconversion in 1 of 5, 6 of 12 and 13 of 13 qPCR-confirmed SARS-CoV-2-infected individuals at 1–5, 6–14 or > 14 days after disease onset, respectively (Figure 1A). As expected, we observed a correlation between binding and neutralisation (Figure 1B). Of note, we also observed that some samples with low S-binding capacity enhanced the infection, as previously described [22].

A. Single point validation of S ELISA set-up. Shown are samples from qPCR-confirmed COVID-19 patients 1–5 days post-symptom onset (n = 5), 6–14 days (n = 12), > 14 days (n = 13), hCoV (n = 20), other viral infections (n = 55), rheumatoid arthritis (RA) (n = 9), and healthy controls (n = 43). Dotted lines indicate the threshold to define a positive sample (OD = 0.7).

B. Correlation between S ELISA and microneutralisation at serum dilution 1/50 for qPCR confirmed SARS-COV-2 positive individuals (n=30) sampled 2-30 days post-symptom onset.

C. Comparison between S ELISA and Abbott Architect SARS-CoV-2 IgG (N) for detection of SARS-CoV-2 infection using serum from qPCR confirmed COVID-19 patients (n = 23) and serum from patients with other viral infections and healthy controls (n = 30). Blue circles indicate individuals positive for anti-S IgG in the S ELISA test. Dotted line indicate threshold to define a positive sample (OD: 0.7).

D. Competition ELISA where remaining anti-S reactivity was measured in paired serum samples after pre-incubation with soluble S-2P protein to remove S-directed antibodies, or with PBS as a negative control.

We compared our S-ELISA assay with the Abbott Architect SARS-CoV-2 IgG and LIASON SARS-CoV-2 S1/S2 that were the standard assays for SARS-CoV-2 serology at the Clinical Microbiology Laboratory at the University Hospital of Umeå by using the samples mentioned in the methods section. Our assay had markedly increased sensitivity to detect low levels of S-specific IgG in serum samples, as shown in Supplementary Figure S1B–D. As a final test of sensitivity, we screened 145 serum samples for the presence of anti-S IgG. Here, we observed seroconversion to anti-S IgG in 17 of 145 (11.7%) individuals, whereas the Abbot Architect SARS-CoV-2 assay only detected seroconversion to anti-N IgG in 10 (6.8%) individuals (Figure 1C). Since binding to S-2P in serum from the 7 discrepant samples was abrogated by pre-incubation with an excess of soluble S-2P (Figure 1D), we concluded that the seven discrepant samples were from de facto SARS-CoV-2 exposed individuals.

### Clinical assay validation on capillary blood samples 

**Figure 2:**
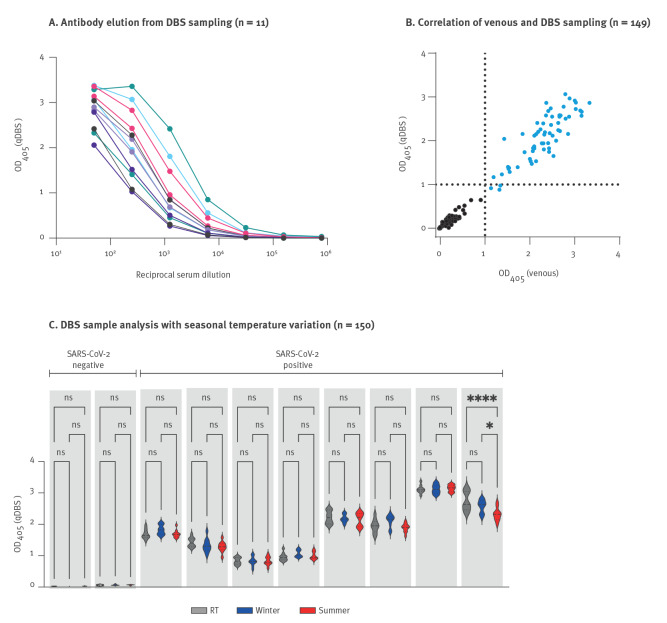
Verification of at-home capillary blood self-sampling test methodology, Region Västerbotten, Sweden, September–November 2020

COVID-19: coronavirus disease; DBS: dried blood spot; RT: room temperature; SARS-CoV-2: severe acute respiratory syndrome coronavirus 2.

After validating our assay on venous blood samples in a clinical setting, it was implemented in May 2020 as a benchmark assay at Region Västerbotten for serological assessment of previous SARS-CoV-2 exposure. We clinically validated the S-ELISA assay by comparing parallel venous and qDBS blood sampling from 149 individuals. We detected 65 serum samples positive for anti-S IgG and 85 negative samples by S-ELISA. The results verified that S-specific antibodies could be eluted from the qDBS, and that these could bind S-2P in a concentration-dependent manner (Figure 2A). In addition, we found a strong linear relationship between OD values between venous and qDBS sampling (r^2^ = 0.96; p < 0.0001, Figure 2B), and that binding was specific to S-2P by competition ELISA, as shown in Supplementary Figure S2A. 

A. The binding of antibodies extracted from 11 individuals sampled with qDBS sample cards to S-2P in concentrations 1/50 to 1/718,250.

B. Comparison of ELISA OD readout from venous and matched qDBS sample cards from 145 individuals, where 65 were defined as SARS-CoV-2-experienced (blue dots) and 84 as naive (black dots).

C. Analysis of plasma from SARS-COV-2 positive (n = 8) and negative (n = 2) individuals extracted from qDBS cards (n = 150) stored under conditions that mimic extreme summer (red, n = 50) or winter (black, n = 50), and RT (grey, n = 50).

We used non-parametric Kruskal-Wallis test for comparisons and Dunn’s multiple comparison test for post-hoc analyses. Significance is indicated as follows: * p < 0.05 and **** p < 0.0001.

To investigate the possibility of using qDBS sample cards for at-home-sampling, we then tested if samples were affected by storage under summer or winter conditions before analysis. This was done by analysis of S-specific IgG from qDBS sample cards that had been incubated under artificial winter or summer conditions for 56h. The qDBS sample cards had been spiked with SARS-CoV-2 positive (n = 8) or negative plasma (n = 2). Results from this experiment demonstrated that all positive samples were well above background and that results were similar for seven out of eight individual samples, where the qDBS had been incubated under summer conditions (Figure 2C). 

As a final test, we performed a survey to understand the ease by which qDBS blood sampling could be managed by unexperienced individuals (n = 62). In this survey, we found that two of 59 individuals reported difficulties with understanding how to perform the sampling and five of 62 individuals did not succeed with the sampling, as shown in Supplementary Figure S2B.

###  At-home testing of SARS-CoV-2 serology by self-sampling in a rural region of Sweden

**Figure 3:**
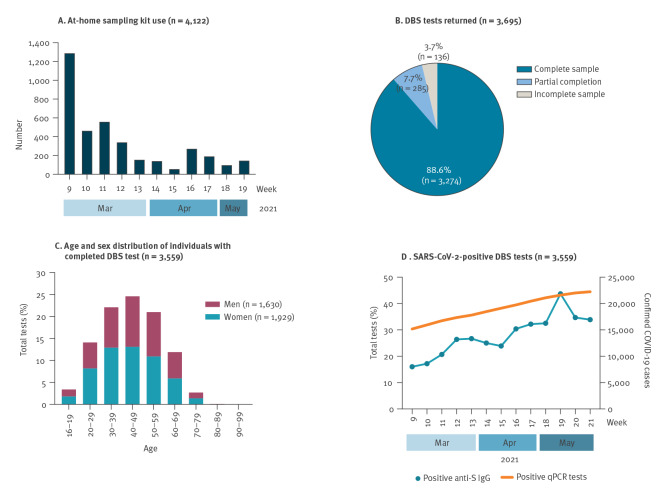
At-home SARS-CoV-2 serology testing of the population using dried blood spot sampling kits in Region Västerbotten, Sweden, March−May 2021 (n = 4,122)

COVID-19: coronavirus disease; DBS: dried blood spot; SARS-CoV-2: severe acute respiratory syndrome coronavirus 2.

During March through May 2021, 4,122 individual tests were distributed throughout Region Västerbotten and 90% (n = 3,695) were returned to the laboratory for analysis (Figure 3A). Of these, 96.3% (n = 3,559) were of adequate quality for elution of proteins from the dried blood spots (Figure 3B). Slightly more women than men were represented (45.8% men, n = 1,630, 54.2% women, n = 1,929) and most tests had been requested from individuals between 30 and 59 years of age (Figure 3C). During the test period, there was an increase of individuals testing positive for SARS-CoV-2 infection by qPCR. Consistently, we could demonstrate an increase in frequency of S-positive qDBS samples that had been analysed (Figure 3D).

A. Number of qDBS tests distributed over time in Region Västerbotten during March to May 2021.

B. Pie chart representing the success rate of qDBS tests returned for analysis. The dark blue field represents the amount of qDBS cards returned with a complete sample (2/2 chambers filled with blood). The light blue represents cards with partial completion (1/2 chambers filled with blood). The light grey area represents an incomplete sample which failed (0/2).

C. Age and sex distribution of successfully completed qDBS tests.

D. The fraction of serological tests positive for anti-S IgG (blue dotted line) and the accumulated number of SARS-CoV-2-positive qPCR tests (orange line) in Region Västerbotten during the same period of time (data Region Västerbotten [35].

**Figure 4:**
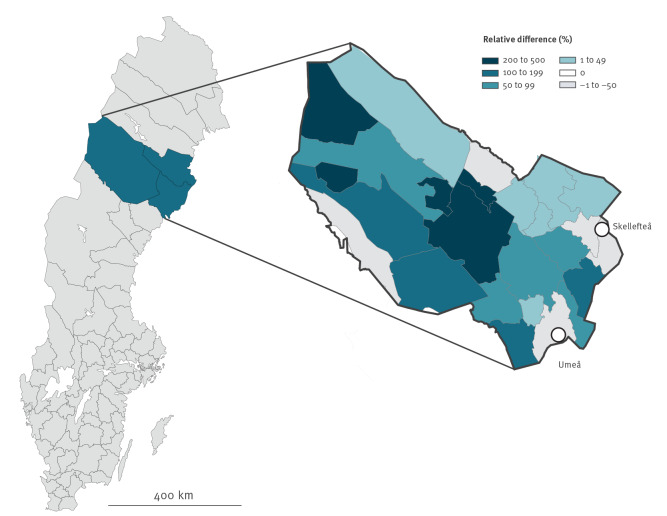
Access to SARS-CoV-2 serological testing in Region Västerbotten, Sweden, by venous blood sampling, November 2020–January 2021 (n = 8,002) and dried blood spot self-sampling, March−May 2021 (n = 4,122)

During November 2020 through January 2021, a total of 8,002 venous blood draws had been performed at healthcare centres or mobile sampling centres in Region Västerbotten. This allowed us to compare the geographical distribution of venous blood sampling with at-home-sampling. We found that 26.3% (n = 2,107) of the venous samples and 44.4% (n = 1,831) of the capillary samples originated from individuals living in the rural areas of Västerbotten. By this, we conclude that the at-home-sampling strategy had resulted in an increased proportion of tests in rural areas while the strategy of venous blood sampling was skewed towards the two major urban areas of Umeå and Skellefteå (Figure 4).

The map inset of Sweden shows Västerbotten subdivided in geographical area codes. The colour distribution shows the over or under representation of at-home testing using quantitative dried blood spots (qDBS) compared to venous testing for serology. The two urban areas of Umeå and Skellefteå are indicated. The large map is subdivided in major administrative regions.

## Discussion

In this study, we outline the development and clinical implementation of a sensitive method for detection of previous SARS-CoV-2 infection and the further development of this assay for at-home sampling. The rationale for the latter was to increase access to high quality diagnostics for all individuals living within a large but sparsely populated region in the northern Sweden.

In 2021, the Swedish Public Health agency performed a head-to-head comparison of our assay (denominated ‘in house Region Västerbotten’) against 11 commercially available assays. There, they found that in house Region Västerbotten had the highest sensitivity and specificity of all assays tested [23]. For this reason, the assay was subsequently used on venous blood samples for probabilistic classification of anti-SARS-CoV-2 antibody responses [24].

When applying eluates from qDBS sample cards instead of serum from venous blood draws, we could demonstrate a high correlation between the sampling methods. This adds to data were other DBS strategies have been used to survey for SARS-CoV-2 exposure [25,26]. Importantly, the detection of S-specific IgG from qDBS elutes were minimally affected by conditions that mimic climate variations in northern Sweden. Here, the strategy was implemented in clinical care and over 4,000 tests were distributed throughout the region and then analysed at the Clinical Microbiology Laboratory at the University Hospital of Umeå.

The strategy outlined herein has several benefits to that of serological analysis by venous blood sampling. These include sampling that do not require trained professionals, outreach to geographically challenging areas and reduced risk for spread of SARS-CoV-2 upon sampling. Moreover, the use of qDBS allows for downstream processing and implementation of tests of with high specificity and sensitivity [27-29]. Because of these benefits and the scalability of the strategy, it was subsequently implemented by the Swedish Public Health Agency for serosurveillance studies to determine country-wide exposure or vaccination during 2021 and 2022 [30,31]. Remote sampling as a methodology is possible to utilise for other purposes than serosurveillance. Examples include monitoring of therapy with measuring of blood concentrations of pharmaceuticals, drug screening and serological diagnostics. Considering the low population density in northern Sweden, the workflow described herein is thus attractive on many levels. It is also applicable to many other regions in Europe with similar challenges in health care outreach to the population. The use of capillary blood sampling for large-scale serological studies is not limited to SARS-CoV-2 S protein, and the feasibility of the strategy for detection of antibodies to other antigens has previously been demonstrated [26,32-34]. It is easy to envision a comprehensible national surveillance system of vaccine effectiveness, especially focussed upon populations that receive care outside of centralised hospital facilities, such as vulnerable and frail individuals at nursing homes or long-term care facilities. The findings herein facilitate the implementation of capillary sampling for such programs.

Our study has some limitations. The goal of the study was to investigate the potential of using at-home-sampling for serological studies in a rural region. Except for the COVID-19 samples used for optimisation of our assay, we did not have data on time of infection with SARS-CoV-2 for any of the cohorts studied here. Also, we did not have access to data concerning disease severity or comorbidities in the larger population cohort. Moreover, we only studied one distinct time period. Collectively, this prohibited a more thorough analysis of the relationship between anti-S IgG and time from exposure to SARS-CoV-2, or development of population-based immune responses to SARS-CoV-2 over time. In the absence of such data, we did not determine how a SARS-CoV-2 N-based ELISA compared with the S-based responses we measured here.

## Conclusions

We here present a strategy for serological surveillance that was implemented for serosurveillance of SARS-CoV-2 infection by at-home sampling. We show that method can be implemented in clinical care even during winter season in arctic or sub-artic areas. Importantly, we show that at-home testing in combination with sensitive methodology reduces geographical inequalities for access to healthcare in rural regions. This, in the end, provides less biased data to inform public health policy.

## Supplementary Data

863000 Supplement

## References

[1] Milne G Hames T Scotton C Gent N Johnsen A Anderson RM Does infection with or vaccination against SARS-CoV-2 lead to lasting immunity? Lancet Respir Med. 2021;9(12):1450-66. 10.1016/S2213-2600(21)00407-0 34688434 PMC8530467

[2] Altarawneh HN Chemaitelly H Hasan MR Ayoub HH Qassim S AlMukdad S Protection against the Omicron variant from previous SARS-CoV-2 infection. N Engl J Med. 2022;386(13):1288-90. 10.1056/NEJMc2200133 35139269 PMC8849180

[3] Folkhälsomyndigheten (FoHM). Teststrategi för covid-19 under 2021. [Testing strategy för covid-19 during 2021]. Stockholm: FoHM. [Accessed: 9 Feb 2023]. Swedish. Available from: https://www.folkhalsomyndigheten.se/contentassets/a1c9634c15dd487e8a8575ff10a53319/teststrategi-for-covid-19-under-2021.pdf

[4] Michlmayr D Hansen CH Gubbels SM Valentiner-Branth P Bager P Obel N Observed protection against SARS-CoV-2 reinfection following a primary infection: A Danish cohort study among unvaccinated using two years of nationwide PCR-test data. Lancet Reg Health Eur. 2022;20:100452. 10.1016/j.lanepe.2022.100452 35791335 PMC9245510

[5] Hansen CH Friis NU Bager P Stegger M Fonager J Fomsgaard A Risk of reinfection, vaccine protection, and severity of infection with the BA.5 omicron subvariant: a nation-wide population-based study in Denmark. Lancet Infect Dis. 2023;23(2):167-76. 10.1016/S1473-3099(22)00595-3 36270311 PMC9578720

[6] Goldberg Y Mandel M Bar-On YM Bodenheimer O Freedman LS Ash N Protection and waning of natural and hybrid immunity to SARS-CoV-2. N Engl J Med. 2022;386(23):2201-12. 10.1056/NEJMoa2118946 35613036 PMC9165562

[7] The Lancet Infectious Diseases . Why hybrid immunity is so triggering. Lancet Infect Dis. 2022;22(12):1649. 10.1016/S1473-3099(22)00746-0 36372089 PMC9648977

[8] Krammer F Simon V . Serology assays to manage COVID-19. Science. 2020;368(6495):1060-1. 10.1126/science.abc1227 32414781

[9] Lopman BA Shioda K Nguyen Q Beckett SJ Siegler AJ Sullivan PS A framework for monitoring population immunity to SARS-CoV-2. Ann Epidemiol. 2021;63:75-8. 10.1016/j.annepidem.2021.08.013 34425208 PMC8379082

[10] Shioda K Lau MSY Kraay ANM Nelson KN Siegler AJ Sullivan PS Estimating the cumulative incidence of SARS-CoV-2 infection and the infection fatality ratio in light of waning antibodies. Epidemiology. 2021;32(4):518-24. 10.1097/EDE.0000000000001361 33935138 PMC8162228

[11] To KK Tsang OT Leung WS Tam AR Wu TC Lung DC Temporal profiles of viral load in posterior oropharyngeal saliva samples and serum antibody responses during infection by SARS-CoV-2: an observational cohort study. Lancet Infect Dis. 2020;20(5):565-74. 10.1016/S1473-3099(20)30196-1 32213337 PMC7158907

[12] Long QX Liu BZ Deng HJ Wu GC Deng K Chen YK Antibody responses to SARS-CoV-2 in patients with COVID-19. Nat Med. 2020;26(6):845-8. 10.1038/s41591-020-0897-1 32350462

[13] Wang Y Wang Y Chen Y Qin Q . Unique epidemiological and clinical features of the emerging 2019 novel coronavirus pneumonia (COVID-19) implicate special control measures. J Med Virol. 2020;92(6):568-76. 10.1002/jmv.25748 32134116 PMC7228347

[14] Ing AJ Cocks C Green JP . COVID-19: in the footsteps of Ernest Shackleton. Thorax. 2020;75(8):693-4. 10.1136/thoraxjnl-2020-215091 32461231 PMC7402559

[15] Beyerl J Rubio-Acero R Castelletti N Paunovic I Kroidl I Khan ZN A dried blood spot protocol for high throughput analysis of SARS-CoV-2 serology based on the Roche Elecsys anti-N assay. EBioMedicine. 2021;70:103502. 10.1016/j.ebiom.2021.103502 34333234 PMC8320407

[16] Swedish Ministry of Social Affairs. Ökad nationell testning för covid-19, 2020 - Överenskommelse mellan staten och Sveriges Kommuner och Regioner. DNR: S2020/05276. [Increased national testing for covid-19, 2020 - Agreement between the state and The Swedish Association of Local Authorities and Regions]. Stockholm: Swedish Government; 2020. Swedish. Available from: https://www.regeringen.se/overenskommelser-och-avtal/2020/06/okad-nationell-testning-for-covid-19-2020---overenskommelse-mellan-staten-och-sveriges-kommuner-och-regioner

[17] Folkhälsomyndigheten (FoHM). Nationell plan för vaccination mot covid-19. [National plan for vaccination against COVID-19]. Stockholm: FoHM; 2020. Swedish. Available from: https://www.folkhalsomyndigheten.se/contentassets/d4c81c0ca7814f79a61bb457d4baab49/nationell-plan-vaccination-covid-19-rekommendation-prioritering.pdf

[18] Wrapp D Wang N Corbett KS Goldsmith JA Hsieh CL Abiona O Cryo-EM structure of the 2019-nCoV spike in the prefusion conformation. Science. 2020;367(6483):1260-3. 10.1126/science.abb2507 32075877 PMC7164637

[19] Hober S Hellström C Olofsson J Andersson E Bergström S Jernbom Falk A Systematic evaluation of SARS-CoV-2 antigens enables a highly specific and sensitive multiplex serological COVID-19 assay. Clin Transl Immunology. 2021;10(7):e1312. 10.1002/cti2.1312 34295471 PMC8288725

[20] United States Food and Drug Administration. (US FDA). Policy for Coronavirus Disease-2019 Tests - Guidance for Developers and Food and Drug Administration Staff. 4th edition. Silver Spring, Maryland, US: USFDA; 2020. Available from: https://www.fda.gov/regulatory-information/search-fda-guidance-documents/policy-coronavirus-disease-2019-tests-during-public-health-emergency-revised

[21] Amanat F Stadlbauer D Strohmeier S Nguyen THO Chromikova V McMahon M A serological assay to detect SARS-CoV-2 seroconversion in humans. Nat Med. 2020;26(7):1033-6. 10.1038/s41591-020-0913-5 32398876 PMC8183627

[22] Okuya K Hattori T Saito T Takadate Y Sasaki M Furuyama W Multiple Routes of Antibody-Dependent Enhancement of SARS-CoV-2 Infection. Microbiol Spectr. 2022;10(2):e0155321. 10.1128/spectrum.01553-21 35319248 PMC9045191

[23] Lagerqvist N Maleki KT Verner-Carlsson J Olausson M Dillner J Wigren Byström J Evaluation of 11 SARS-CoV-2 antibody tests by using samples from patients with defined IgG antibody titers. Sci Rep. 2021;11(1):7614. 10.1038/s41598-021-87289-6 33828214 PMC8027209

[24] Castro Dopico X Muschiol S Grinberg NF Aleman S Sheward DJ Hanke L Probabilistic classification of anti-SARS-CoV-2 antibody responses improves seroprevalence estimates. Clin Transl Immunology. 2022;11(3):e1379. 10.1002/cti2.1379 35284072 PMC8891432

[25] Meyers E Coen A De Sutter A Padalko E Callens S Vandekerckhove L Diagnostic performance of the SARS-CoV-2 S1RBD IgG ELISA (ImmunoDiagnostics) for the quantitative detection of SARS-CoV-2 antibodies on dried blood spots. J Clin Virol. 2022;155:105270. 10.1016/j.jcv.2022.105270 36027822 PMC9388275

[26] Wong MP Meas MA Adams C Hernandez S Green V Montoya M Development and implementation of dried blood spot-based COVID-19 serological assays for epidemiologic studies. Microbiol Spectr. 2022;10(3):e0247121. 10.1128/spectrum.02471-21 35612315 PMC9241704

[27] Klumpp-Thomas C Kalish H Drew M Hunsberger S Snead K Fay MP Standardization of ELISA protocols for serosurveys of the SARS-CoV-2 pandemic using clinical and at-home blood sampling. Nat Commun. 2021;12(1):113. 10.1038/s41467-020-20383-x 33397956 PMC7782755

[28] Toh ZQ Higgins RA Anderson J Mazarakis N Do LAH Rautenbacher K The use of dried blood spots for the serological evaluation of SARS-CoV-2 antibodies. J Public Health (Oxf). 2022;44(2):e260-3. 10.1093/pubmed/fdab011 33611565 PMC7928805

[29] Roxhed N Bendes A Dale M Mattsson C Hanke L Dodig-Crnković T Multianalyte serology in home-sampled blood enables an unbiased assessment of the immune response against SARS-CoV-2. Nat Commun. 2021;12(1):3695. 10.1038/s41467-021-23893-4 34140485 PMC8211676

[30] Beser J Galanis I Enkirch T Kühlmann Berenzon S van Straten E Duracz J Seroprevalence of SARS-CoV-2 in Sweden, April 26 to May 9, 2021. Sci Rep. 2022;12(1):10816. 10.1038/s41598-022-15183-w 35752708 PMC9233662

[31] Folkhälsomyndigheten (FoHM). Undersökningar av förekomsten av antikroppar mot SARS-CoV-2. [Investigations of the presence of antibodies against SARS-CoV-2]. Stockholm: FoHM. [Accessed: 1 May 2022]. Swedish. Available from: https://www.folkhalsomyndigheten.se/smittskydd-beredskap/utbrott/aktuella-utbrott/covid-19/statistik-och-analyser/undersokningar-och-datainsamlingar/forekomst-av-antikroppar

[32] Hergott DEB Owalla TJ Balkus JE Apio B Lema J Cemeri B Feasibility of community at-home dried blood spot collection combined with pooled reverse transcription PCR as a viable and convenient method for malaria epidemiology studies. Malar J. 2022;21(1):221. 10.1186/s12936-022-04239-x 35836179 PMC9284728

[33] Barquín D Ndarabu A Carlos S Fernández-Alonso M Rubio-Garrido M Makonda B HIV-1 diagnosis using dried blood spots from patients in Kinshasa, DRC: a tool to detect misdiagnosis and achieve World Health Organization 2030 targets. Int J Infect Dis. 2021;111:253-60. 10.1016/j.ijid.2021.08.035 34419584

[34] Øverbø J Aziz A Zaman K Julin CH Qadri F Stene-Johansen K Stability and feasibility of dried blood spots for hepatitis E virus serology in a rural setting. Viruses. 2022;14(11):2525. 10.3390/v14112525 36423134 PMC9692628

[35] Region Västerbotten. Aktuellt läge – följ statistik för covid 19. [Current situation – follow statistics for covid-19]. Umeå: Region Västerbotten. [Accessed: 7 Jun 2021]. Swedish. Available from: https://www.regionvasterbotten.se/coronavirus/aktuellt-vardlage-i-region-vasterbotten-covid-19

[36] Cox RJ Brokstad KA . Not just antibodies: B cells and T cells mediate immunity to COVID-19. Nat Rev Immunol. 2020;20(10):581-2. 10.1038/s41577-020-00436-4 32839569 PMC7443809

[37] Grifoni A Weiskopf D Ramirez SI Mateus J Dan JM Moderbacher CR Targets of T cell responses to SARS-CoV-2 coronavirus in humans with COVID-19 disease and unexposed individuals. Cell. 2020;181(7):1489-1501.e15. 10.1016/j.cell.2020.05.015 32473127 PMC7237901

[38] GeurtsvanKessel CH Okba NMA Igloi Z Bogers S Embregts CWE Laksono BM An evaluation of COVID-19 serological assays informs future diagnostics and exposure assessment. Nat Commun. 2020;11(1):3436. 10.1038/s41467-020-17317-y 32632160 PMC7338506

[39] Okba NMA Müller MA Li W Wang C GeurtsvanKessel CH Corman VM Severe acute respiratory syndrome coronavirus 2-specific antibody responses in coronavirus disease patients. Emerg Infect Dis. 2020;26(7):1478-88. 10.3201/eid2607.200841 32267220 PMC7323511

[40] Hoffmann M Kleine-Weber H Schroeder S Krüger N Herrler T Erichsen S SARS-CoV-2 cell entry depends on ACE2 and TMPRSS2 and is blocked by a clinically proven protease inhibitor. Cell. 2020;181(2):271-280.e8. 10.1016/j.cell.2020.02.052 32142651 PMC7102627

[41] Jiang S Hillyer C Du L . Neutralizing antibodies against SARS-CoV-2 and other human coronaviruses. Trends Immunol. 2020;41(5):355-9. 10.1016/j.it.2020.03.007 32249063 PMC7129017

