# Supplementary material for: At-home sampling to meet geographical challenges for serological assessment of SARS-CoV-2 exposure in a rural region of northern Sweden, March to May 2021: a retrospective cohort study
Source: Euro Surveill. 2023 Mar 30;28(13):2200432. doi: 10.2807/1560-7917.ES.2023.28.13.2200432 (PMC10064644; doi:10.2807/1560-7917.ES.2023.28.13.2200432)
Supplement: Supplement [file 22-00432_FORSELL_SUPPLEMENT.pdf]

This supplementary material is hosted by Eurosurveillance as supporting information alongside the article *At-home sampling to meet geographical challenges for serological assessment of SARS-CoV-2 exposure in a rural region of northern Sweden, March to May 2021: a retrospective cohort study*, on behalf of the authors, who remain responsible for the accuracy and appropriateness of the content. The same standards for ethics, copyright, attributions and permissions as for the article apply. Supplements are not edited by Eurosurveillance and the journal is not responsible for the maintenance of any links or email addresses provided therein.

Supplementary Figure S1

Titration of SARS-CoV-2 IgG positive and negative serum, and comparison of in-house AP S-ELISA, Abbot Architecht SARS-CoV-2 N IgG and Liason SARS-CoV-2 S1/S2 IgG methods

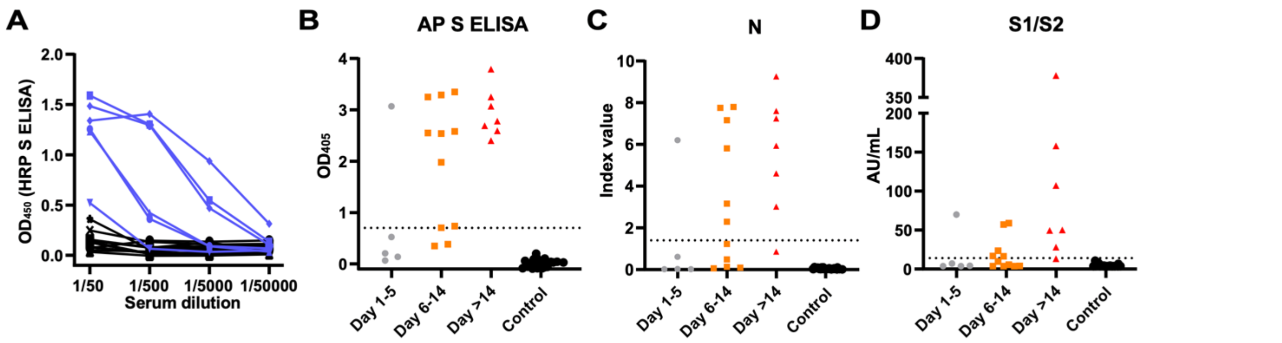

(A) Titration of serum from qPCR confirmed Covid-19 positive individuals (N=6, blue) and negative control samples (N=11, black). Side by side validation between **(B)** in-house AP S-ELISA, **(C)** Abbott Architect 1 SARS-CoV-2 N IgG and **(D)** LIAISON® SARS-CoV-2 S1/S2 IgG methods.

Supplementary Figure S2

S-2P Specific binding by competition ELISA, and qDBS at-home sampling survey results

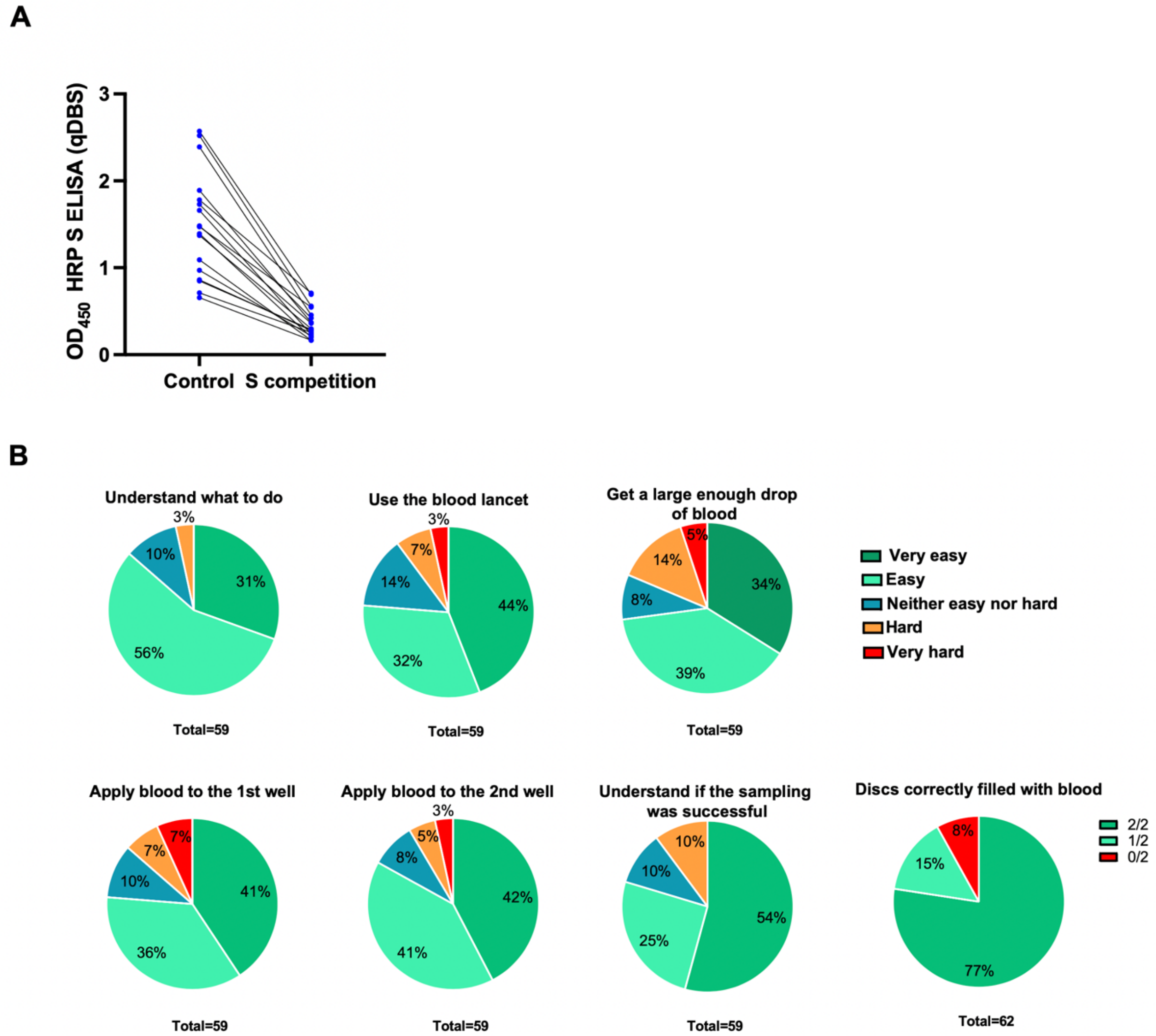

(A) Detection of anti-S IgG in qDBS elutes after co-incubation with an excess of soluble S-2P protein. **(B)** Evaluation of the ease of self-sampling with the provided self-sampling kit.
